# A Nematode Calreticulin, Rs-CRT, Is a Key Effector in Reproduction and Pathogenicity of *Radopholus similis*

**DOI:** 10.1371/journal.pone.0129351

**Published:** 2015-06-10

**Authors:** Yu Li, Ke Wang, Hui Xie, Yan-Tao Wang, Dong-Wei Wang, Chun-Lin Xu, Xin Huang, De-Sen Wang

**Affiliations:** 1 Laboratory of Plant Nematology and Research Center of Nematodes of Plant Quarantine, Department of Plant Pathology, South China Agricultural University, Guangzhou, Guangdong, 510642, China; 2 Paulownia Research and Development Center of State Forestry Administration, Zhengzhou, Henan, 450003, China; 3 Department of Entomology, Rutgers University, New Brunswick, New Jersey, 08901, United States of America; Institute of Genetics and Developmental Biology, Chinese Academy of Sciences, CHINA

## Abstract

*Radopholus similis* is a migratory plant-parasitic nematode that causes severe damage to many agricultural and horticultural crops. Calreticulin (CRT) is a Ca^2+^-binding multifunctional protein that plays key roles in the parasitism, immune evasion, reproduction and pathogenesis of many animal parasites and plant nematodes. Therefore, CRT is a promising target for controlling *R*. *similis*. In this study, we obtained the full-length sequence of the CRT gene from *R*. *similis *(*Rs-crt*), which is 1,527-bp long and includes a 1,206-bp ORF that encodes 401 amino acids. *Rs*-CRT and *Mi*-CRT from *Meloidogyne incognita* showed the highest similarity and were grouped on the same branch of the phylogenetic tree. *Rs-crt* is a multi-copy gene that is expressed in the oesophageal glands and gonads of females, the gonads of males, the intestines of juveniles and the eggs of *R*. *similis*. The highest *Rs-crt* expression was detected in females, followed by juveniles, eggs and males. The reproductive capability and pathogenicity of *R*. *similis* were significantly reduced after treatment with *Rs-crt* dsRNA for 36 h. Using plant-mediated RNAi, we confirmed that *Rs-crt* expression was significantly inhibited in the nematodes, and resistance to *R*. *similis *was significantly improved in transgenic tomato plants. Plant-mediated RNAi-induced silencing of *Rs-crt* could be effectively transmitted to the F2 generation of *R*. *similis*; however, the silencing effect of *Rs-crt* induced by *in vitro* RNAi was no longer detectable in F1 and F2 nematodes. Thus, *Rs-crt* is essential for the reproduction and pathogenicity of *R*. *similis*.

## Introduction

The burrowing nematode, *Radopholus similis* [(Cobb, 1893) Thorne, 1949] is a migratory plant parasitic nematode, that has been listed as a quarantine plant pest in many countries and regions [1, 2]. *R*. *similis* is known to reproduce on more than 250 plant species [3] and causes severe economic losses [4–6]. At present, the inability to control *R*. *similis* is a worldwide problem. Therefore, it is of particular importance to study and establish effective methods for controlling *R*. *similis*.

RNA interference (RNAi), which was first discovered in *Caenorhabditis elegans*, has been shown to be effective in most eukaryotic organisms [7]. RNAi mediated by dsRNA soaking (*in vitro* RNAi) has been developed as an effective tool for studying gene function in many plant nematodes and other organisms [8–13]. However, the silencing effect of *in vitro* RNAi on some genes in plant-parasitic nematodes is highly time-limited [11, 14]. As an alternative, plant-mediated RNAi has been successfully applied for gene knockdown with the aim of studying gene function and controlling many pests that impact crop production [15–20]. Plant-mediated RNAi may likewise serve as an effective technique for controlling plant nematodes in agriculture. However, most previous studies have focused on sedentary plant endoparasitic nematodes [21–26], and there is limited information available regarding the use of plant-mediated RNAi to control migratory plant parasitic nematodes.

Calreticulin (CRT) is a Ca^2+^-binding multifunctional protein that is highly conserved in animals and plants [27]. CRT has been shown to be secreted into host tissues by many animal parasites and to play important roles in skin penetration, infection, parasitism, pathogenesis and immune evasion [28–33]. In *C*. *elegans*, CRT is required for the stress response and for fertility [34]. In plant parasitic nematodes, CRT has only been isolated from the oesophageal secretions of *Meloidogyne incognita* [35–37], *Ditylenchus destructor* (GenBank accession number ACV33082) and *Bursaphelenchus xylophilus* [38].

In plant parasitic nematodes, oesophageal secretions (also referred to as stylet secretions) are produced by oesophageal subventral and dorsal glands cells and are secreted through the stylet during parasitism[39]. These stylet secretions are thought to play key roles throughout the process of parasitism [39–41]. At present, in addition to the calreticulin gene (*crt*), a variety of genes expressed in oesophageal gland cells have been identified and studied in plant nematodes. It has been reported that CRT plays important roles in parasitism and the suppression of plant defences in *M*. *incognita* [36, 37, 39] and in the reproduction of *B*. *xylophilus* [38]. As a promising target for controlling parasites, CRT has attracted much attention. However, the functions of the calreticulin gene in *R*. *similis* (*Rs-crt*) have not yet been examined. In this study, *Rs-crt* was first cloned from *R*. *similis*, and its structure and features were analyzed. The localization and expression of *Rs-crt* were determined in *R*. *similis* through *in situ* hybridization and qPCR, and the role of *Rs-crt* in reproduction and pathogenesis was investigated using *in vitro* RNAi and plant-mediated RNAi. In addition, we obtained transgenic tomato plants expressing *Rs-crt* dsRNA that showed obvious resistance to *R*. *similis*.

## Materials and Methods

### Ethics statement

We collected the nematodes in areas where banana-burrowing nematodes occured and no specific permits were required. The land used as the collection area is neither privately owned nor protected in any way, and the field studies did not involve endangered or protected species.

### Nematode cultivation and extraction

*R*. *similis* was collected from the roots of *Anthurium andraeanum*, an ornamental plant, and cultured on carrot disks at 25°C as previously described [42]. At 50 d after inoculation, the cultured nematodes were extracted from the carrot disks as described elsewhere [12].

### Plant materials

Tomato seeds were purchased from Changhe, Guangzhou, and surface sterilized as previously described [20]. The sterilized seeds were sown in 1.5 L of sterilized soil and grown in a greenhouse for 30 d. The sterilized seeds were also germinated on half-strength MS medium (pH 5.8) with 0.3% Phytagel [43] and cultured in a 25°C chamber (16 h-light/8 h-dark cycle) [20].

### Cloning of *Rs-crt* from *R*. *similis*

Total RNA and genomic DNA (gDNA) were extracted from 20,000 mixed-stage nematodes using TRIzol reagent (Invitrogen, Carlsbad, CA, USA) and a tissue DNA kit (Magen, China), respectively. A partial *Rs-crt* cDNA sequence was amplified using the degenerate primers Cal1F and Cal2R (Table 1), which target *crt* from *M*. *incognita* [35]. Based on the sequence of the obtained fragment, 3′ RACE primers (Rscrt-3F/Outer primer) and 5′ RACE primers (SL/Rscrt-5R) (Table 1) [44] were synthesized to amplify the cDNA sequence of *Rs-crt* using the SMART RACE cDNA amplification kit (Clontech, Japan). The purified PCR products (5′ end, middle fragment and 3′ end) were sequenced and spliced into the complete sequence. Based on the spliced complete sequence, two pairs of primers, Rscrt-FullF/Rscrt-FullR and Rscrt-cdsF/Rscrt-cdsR (Table 1), were designed to amplify the full-length cDNA and gDNA sequences of *Rs-crt*.

**Table 1:** Primers used in this study.

Primer name	Sequence	Primer use
Cal1F	5′- GAAGTCTTYTTCAARGAGGAG -3′	PCR amplification
Cal2R	5′- GTTSTCAATYTCTGGRTGKATCCA -3′	PCR amplification
Rscrt-3F	5′- ACAAAGCAAAGAACCACCTGAT -3′	3′- RACE
Outer primer	5′- TACCGTCGTTCCACTAGTGATTT -3′	3′- RACE
SL	5′- GGTTTAATTACCCAAGTTTGAG -3′	5′- RACE
Rscrt-5R	5′- TAACCTTCACATAGCCTCCTCC -3′	5′- RACE
Rscrt-FullF	5′- GGTTTAATTACCCAAGTTTG -3′	cDNA amplification
Rscrt-FullR	5′- CTTGATGTCTGTGTCCATTCC -3′	cDNA amplification
Rscrt-cdsF	5′- GGAAATGATTAAATCAGTTGCACTC -3′	DNA amplification
Rscrt-cdsR	5′- TCTGCTTGTCGGTCTCAGAGCT -3′	DNA amplification
Southern-F	5′- ATCTGTGGCCCTGGTACTAAA -3′	Southern blot
Southern-R	5′- CCATTCTCCGTCCATCTCGT -3′	Southern blot
ISH-T7S^a^	5′- GGATCCTAATACGACTCACTATAGGGggaggctatgtgaaggtta -3′	ISH template
ISH-A	5′- CGGCAGTAGTTCCCAAT -3′	ISH template
ISH-S1	5′- ggaggctatgtgaaggtta-3′	ISH template
ISH-T7A1^a^	5′- GGATCCTAATACGACTCACTATAGGGCGGCAGTAGTTCCCAAT-3′	ISH template
qPCR-F	5′- aagcaaagaaccacctga -3′	qPCR
qPCR-R	5′- CGGCAGTAGTTCCCAAT -3′	qPCR
Actin-F	5′- GAAAGAGGGCCGGAAGAG -3′	qPCR
Actin-R	5′- AGATCGTCCGCGACATAAAG -3′	qPCR
Rscrt-T7S^a^	5′- GGATCCTAATACGACTCACTATAGGGatcgtaagggctgaggtatt-3′	dsRNA template
Rscrt-A	5′- CAGGTGGTTCTTTGCTTTGTA-3′	dsRNA template
Rscrt-S	5′- atcgtaagggctgaggtatt-3′	dsRNA template
Rscrt-T7A^a^	5′-GGATCCTAATACGACTCACTATAGGGACCGTTGCATCCCTGGCT -3′	dsRNA template
eGFP-T7S^a^	5′-GGATCCTAATACGACTCACTATAGGGCAGTGCTTCAGCCGCTACC-3′	dsRNA template
eGFP-A	5′-AGTTCACCTTGATGCCGTTCTT-3′	dsRNA template
eGFP-S	5′-CAGTGCTTCAGCCGCTACC-3′	dsRNA template
eGFP-T7 A^a^	5′- GGATCCTAATACGACTCACTATAGGGAGTTCACCTTGATGCCGTTCTT-3′	dsRNA template
RNAi-F	5′- CCGCTCGAGTCTAGAATCTGTGGCCCTGGTACTAAA -3′	Vector construction
RNAi-R	5′- CTAGCCATGGATCCCCATTCTCCGTCCATCTCGT-3′	Vector construction
eGFP-F	5′-CCGCTCGAGTCTAGATGCTTCAGCCGCTACCC-3′	Vector construction
eGFP-R	5′-CATGCCATGGATCCAGTTCACCTTGATGCCGTTC-3′	Vector construction
CHSA-F	5′- ACTTGCCTTGGAGTTTATGTT -3′	PCR detection
OCS-R	5′- TTGTTATTGTGGCGCTCTATC -3′	PCR detection

^a^ The T7 promoter sequence is underlined.

### Sequence analysis, alignment and phylogenetic analyses

Sequence homology comparisons were conducted using the BLASTX and BLASTN programs from NCBI (http://blast.ncbi.nlm.nih.gov/Blast.cgi). The ORF finder program was employed to predict the open reading frame (http://www.ncbi.nlm.nih.gov/gorf/gorf.html). The protein transmembrane regions, molecular weight, theoretical isoelectric point, glycosylation sites and hydrophobicity sites were predicted using the Protein Machine software available at ExPASy (http://www.expasy.ch/tools/). The signal peptide for secretion and the cleavage site were predicted using the SignalP 3.0 server [45]. The amino acid (aa) sequences of *Rs*-CRT (AFK76483) and six other CRT proteins from *M*. *incognita* (AAL40720), *C*. *elegans* (NP_504575), *Necator americanus* (CAA07254), *D*. *destructor* (ACV33082), *B*. *xylophilus* (ADD82420) and *Pratylenchus goodeyi* (AIW66697) were aligned using DNAMAN. Based on the aa sequences of 22 CRT proteins from 20 species, a neighbour-joining phylogenetic tree was constructed using MEGA5.0 [46].

### Southern blot hybridization

The Southern-F and Southern-R primers (Table 1) were designed to amplify a 378-bp digoxigenin (DIG)-labelled probe using the PCR DIG Probe Synthesis Kit (Roche, Germany). Approximately 10 μg of *R*. *similis* gDNA was digested with *EcoR* I and *Xba* I. The digested DNA was separated via electrophoresis and then transferred to a Hybond N^+^ membrane (Amersham) [38]. Hybridization and detection were carried out using the Dig High Primer DNA Labeling and Detection Starter Kit I (Roche). An equal amount of gDNA from carrot callus was used as a control.

### Tissue localization and expression of *Rs-crt* in *R*. *similis*

The specific primers ISH-T7S/ISH-A and ISH-S1/ISH-T7A1 (Table 1) were designed to synthesize DIG-labelled sense and antisense RNA probes (294-bp) using DIG RNA labelling mix (Roche). *In situ* hybridization was performed as described elsewhere [13, 47]. After hybridization, the nematodes were examined by microscopy (Nikon 90i).

RNA samples were extracted from 100 eggs, juveniles, females and males using the RNeasy Micro kit (Qiagen, Germany), respectively. Total RNA was then treated and quantified as previously described [13]. The RNA extracted from each sample served as a template for synthesizing cDNA using the iScript cDNA synthesis kit (Bio-Rad, USA). The primers qPCR-F/qPCR-R (Table 1) were designed to assay *Rs-crt* expression. The primers Actin-F/Actin-R (Table 1) were employed to amplify β-actin as a reference gene [48]. qPCR was performed using a CFX-96 qPCR instrument (Bio-Rad) with iTaq Universal SYBR Green Supermix (Bio-Rad). The initial data analysis was carried out using Bio-Rad CFX-96 manager software, which created Ct values and extrapolated the relative levels of PCR products from standard curves. Melt curves were obtained routinely, which allowed the possibility of both contamination and primer dimers to be discounted [12, 13]. All expression experiments were performed in triplicate with three biological replicates.

### Knockdown of *Rs-crt* by soaking with target-specific dsRNA

The primers Rscrt-T7S/Rscrt-A and Rscrt-S/Rscrt-T7A, which contained a T7 promoter (Table 1), were designed to transcribe *Rs-crt* sense and antisense single-stranded RNA (ssRNA) using a T7 Transcription Kit (TOYOBO, Japan). The corresponding dsRNA was synthesized and purified as previously described [13, 49]. Non-endogenous control dsRNA (enhanced green fluorescent protein gene, e*gfp*) was synthesized using the primers eGFP-T7S/eGFP-A and eGFP-S/eGFP-T7A (Table 1).

Approximately 1,000 mixed-stage nematodes were soaked in *Rs-crt* dsRNA solution (2.0 mg/mL) at 25°C for 12 h, 24 h, 36 h and 48 h, respectively. e*gfp* dsRNA solution (2.0 mg/mL) was used as a non-endogenous control. The soaking times for the control were the same as those for the *Rs-crt* dsRNAs. Additionally, untreated nematodes were used as a blank control. Tests were subsequently performed as follows: (1) Total RNA was extracted from the nematodes in each treatment group, and qPCR was performed to analyze the suppression of *Rs-crt* mRNA expression in *R*. *similis*, as described above; (2) the total number of nematodes was calculated after culturing 30 different treated females on carrot disks for 50 d at 25°C.

After treatment with *Rs-crt* dsRNA for 36 h, 1,000 mixed-stage nematodes were inoculated onto each of the selected tomato plantlets, which had been grown under the same conditions. Nematodes treated with e*gfp* dsRNA for 36 h were used as a control. Additionally, untreated nematodes were used as a blank control. These plants were cultivated ina greenhouse as described elsewhere [18]. At 60 d after inoculation, three growth parameters (plant height, fresh shoot weight and fresh root weight) were measured. The nematodes in the rhizosphere were extracted and quantified as previously described [12, 50]. Five biological replicates were performed.

### Vector construction and plant transformation

The RNAi-F/RNAi-R primers (Table 1) were designed to amplify a 407-bp target sequence of *Rs-crt*. The digested PCR products were inserted into the X*ho*I/*Nco*I and *Xba*I/*BamH*I sites of pFGC5941 at inverted repeat sequences to obtain the plant RNAi vector pFGC-RS-crt2, which can generate a hairpin RNAi construct. The non-endogenous control vector pFGC-e*gfp*2 was constructed using the primers eGFP-F/eGFP-R (Table 1). The pFGC-RS-crt2 and pFGC-e*gfp*2 vectors and an empty pFGC5941 vector were transformed into *Agrobacterium tumefaciens* (strain EHA105) via the freeze-thaw method [51]. Tomato transformation was performed according to the methods described by Arshad *et al*. [20], and the transformants were selected on MS medium containing kanamycin. The regenerated transgenic plants were transferred to soil and grown in the greenhouse to obtain seeds.

### Molecular analysis of putative transgenic tomato plants

gDNA was extracted from kanamycin-tolerant T0 *Rs-crt* transgenic tomato leaves using the HiPure Plant DNA Maxi Kit (Magen, China) and was assessed via PCR and sequencing using the CHSA-F/OCS-R and RNAi-F/RNAi-R primers (Table 1). The e*gfp* transgenic plants were similarly checked using the CHSA-F/OCS-R and eGFP-F/eGFP-R primers (Table 1). For Southern blot analysis, 15 μg of gDNA from PCR-positive *Rs-crt* transgenic plants was digested with *EcoR* I and transferred to a Hybond-N^+^ membrane. Hybridization and detection were performed as described above. Equal amounts of gDNA from T0 e*gfp* transgenic plants and empty transformation vector plants were used as controls.

Total RNA was extracted from the leaves of PCR- and Southern-positive *Rs-crt* transgenic plants and assessed via RT-PCR using the RNAi-F/RNAi-R primers (Table 1). Positive *Rs-crt* transgenic plants were cultured to obtain seeds. After approximately 60 d, 20 seeds (T1) collected from a single plant were sown in pots. After 30 d of culture, gDNA was extracted from these T1 transgenic plants and checked via PCR using the RNAi-F/RNAi-R primers (Table 1). Positive T1 plants were cultured to obtain T2 homozygous transgenic plants for further analyses.

### Analysis of transgenic tomato plant resistance

A total of 1,000 mixed-stage nematodes were inoculated onto each of the selected T2 *Rs-crt* transgenic plantlets. All plantlets were grown under the same conditions. At 60 d after inoculation, the resistance of the *Rs-crt* transgenic plants was examined according to the method described in a previous section of this report (“Knockdown of *Rs-crt* by soaking with target-specific dsRNA”). T2 e*gfp* transgenic plants, empty transformation vector plants and wild-type tomato plants inoculated with nematodes were used as controls. Five biological replicates were performed.

### Analysis of *Rs-crt* expression in *R*. *similis* feeding on transgenic plants

Total RNA was extracted from 100 mixed-stage nematodes isolated from T2 *Rs-crt* transgenic tomato roots using the RNeasy Micro Kit (Qiagen). qPCR was performed to analyze *Rs-crt* suppression in *R*. *similis* as described above. Nematodes isolated from T2 e*gfp* transgenic plants, empty transformation vector plants and wild-type tomato plants were used as controls.

### Persistence and inheritance of *Rs-crt* silencing induced by *in vitro* RNAi and plant-mediated RNAi

To determine the transcript recovery of *Rs-crt*, mixed-stage nematodes collected from different sources (treated with *Rs-crt* dsRNA for 36 h and isolated from T2 *Rs-crt* transgenic tomato roots) were washed and maintained in sterile water for 1, 3, 5, 7, 9, 11, 13, and 15 d, respectively. Then, 100 nematodes in each group were collected for qPCR analysis as described above. The remaining nematodes were maintained in water at 25°C, and the water was changed daily [14]. Untreated nematodes were used as a control.

Approximately 100 females (P0 generation) collected from different sources were inoculated onto carrot disks. After being cultured at 25°C for 20 d, the first-generation (F1) juveniles and immature females were extracted from the carrot disks and used for the following experiments: (1) A total of 100 mixed-stage F1 nematodes were used for RNA extraction, and qPCR was performed to analyze *Rs-crt* expression in *R*. *similis* as described above; (2) 30 F1 females were inoculated onto carrot disks and incubated at 25°C for 30 d, and the total number of nematodes was subsequently calculated; and (3) wild-type tomato plantlets were selected as described above, and each plantlet was inoculated with 200 mixed-stage nematodes (F1). At 45 d after inoculation, three growth parameters were measured in these plants. The rhizosphere nematodes were extracted and quantified. Plantlets inoculated with untreated nematodes were used as controls. Five biological replicates were performed.

Approximately 100 F1 females collected from different sources were cultured as described above. The obtained second-generation (F2) juveniles and immature females were used for the following experiments: (1) qPCR was performed to analyze *Rs-crt* expression in F2 nematodes as described above; (2) 30 F2 females from different sources were inoculated onto carrot disks and incubated at 25°C for 30 d, and the number of nematodes was calculated; and (3) these F2 female nematodes respectively were cultured to obtain F3 nematodes. qPCR was used to analyze *Rs-crt* expression in F3 nematodes as described above.

### Statistical analysis

The data collected from the experiments were analyzed using SAS 9.2 (SAS Institute, Cary, NC, USA). All of the data obtained in this study were subjected to one-way analysis of variance (ANOVA) and tested for differences between treatments at the 5% level using Duncan’s Multiple Range Test (DMRT).

## Results

### Cloning and characterization of the *Rs-crt* gene in *R*. *similis*

An 832-bp partial cDNA sequence of *Rs-crt* was amplified via qPCR (S1 Fig). Based on the sequence of this fragment, the full-length cDNA sequence of *Rs-crt* was obtained through RACE-PCR (1,527-bp; JX067552). The *Rs-crt* cDNA was composed of a 52-bp 5′-untranslated region (5′-UTR), a 1,206-bp ORF, and a 269-bp 3′-UTR containing a typical polyadenylation signal (ATTAAA) (S2 Fig). Amplification of *Rs-crt* using gDNA as a template yielded a 1,515-bp fragment (from theATG to the stop codon). Introns were identified by aligning the genomic sequence to the corresponding cDNA sequence. *Rs-crt* gDNA contained six introns and seven exons (S3 Fig).

**Fig 1:**
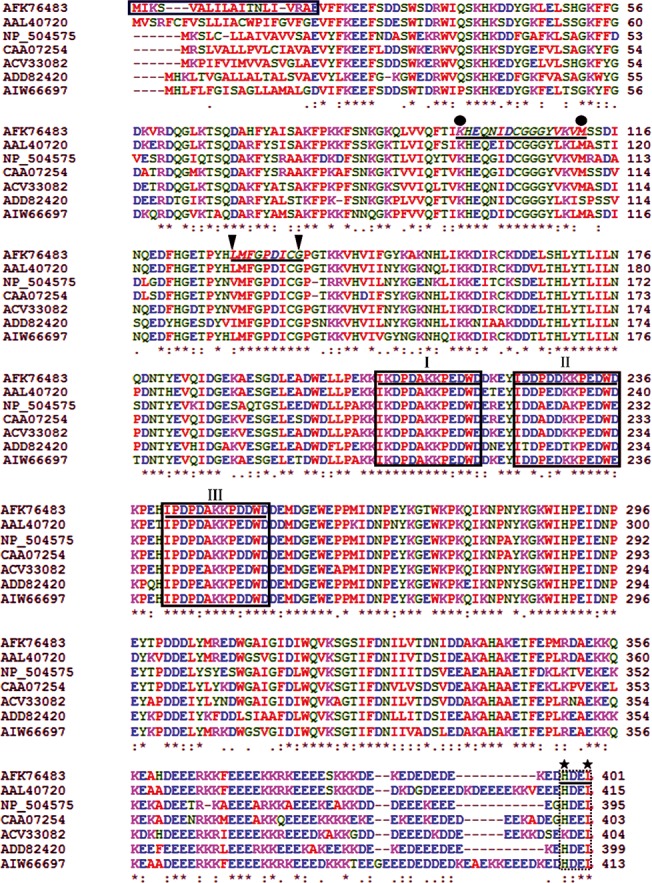
Multiple sequence alignment of the predicted *Radopholus similis Rs*-CRT protein with other nematode CRT proteins. GenBank accession numbers: *R*. *similis* (AFK76483), *Meloidogyne incognita* (AAL40720), *Caenorhabditis elegans* (NP_504575), *Necator americanus* (CAA07254), *Ditylenchus destructor* (ACV33082), *Bursaphelenchus xylophilus* (ADD82420), *Pratylenchus goodeyi* (AIW66697). Box, the putative signal peptide sequences of *Rs*-CRT; I-III, the conserved regions characteristic of the calreticulin class; black circle, possible Ca^2+^-binding signal motif one; black triangle, possible Ca^2+^-binding signal motif two; black pentagram, C-terminal endoplasmic reticulum (ER) retention signal sequences; (*), highly conserved amino acid residues; (:), conserved amino acid residues; (.) conserved cysteine residues.

The *Rs*-CRT protein encodes 401 aa with a theoretical molecular mass of 46.99 kDa and an isoelectric point of 4.73. The molecular formula of *Rs-*CRT is C_2089_H_3175_N_545_O_672_S_10_. An 18 aa signal peptide with a cleavage site between Ala18 and Glu19 was predicted by SignalP 3.0 at the N-terminus of the deduced *Rs*-CRT sequence. BLASTP searches revealed that the *Rs-*CRT aa sequence showed high similarity to CRT sequences from five other nematode species. *Rs*-CRT (AFK76483) had the highest similarity with *Mi*-CRT from *M*. *incognita* (AAL40720, 84% identity and 92% similarity, E-value = 0) and *Pg*-CRT from *P*. *goodeyi* (AIW66697, 84% identity and 92% similarity, E-value = 0). *Rs*-CRT also showed very higher similarity with *Dd*-CRT-1 from *D*. *destructor* (ACV33082, 82% identity and 92% similarity, E-value = 0) and *Bx*-CRT from *B*. *xylophilus* (ADD82420, 73% identity and 84% similarity, E-value = 0). Additionally, *Rs*-CRT exhibited high similarity with *Na*-CRT from *N*. *americanus* (CAA07254, 77% identity and 88% similarity, E-value = 0) and *Ce*-CRT-1 from *C*. *elegans* (NP_504575, 73% identity and 86% similarity, E-value = 0) (Fig 1).

**Fig 2:**
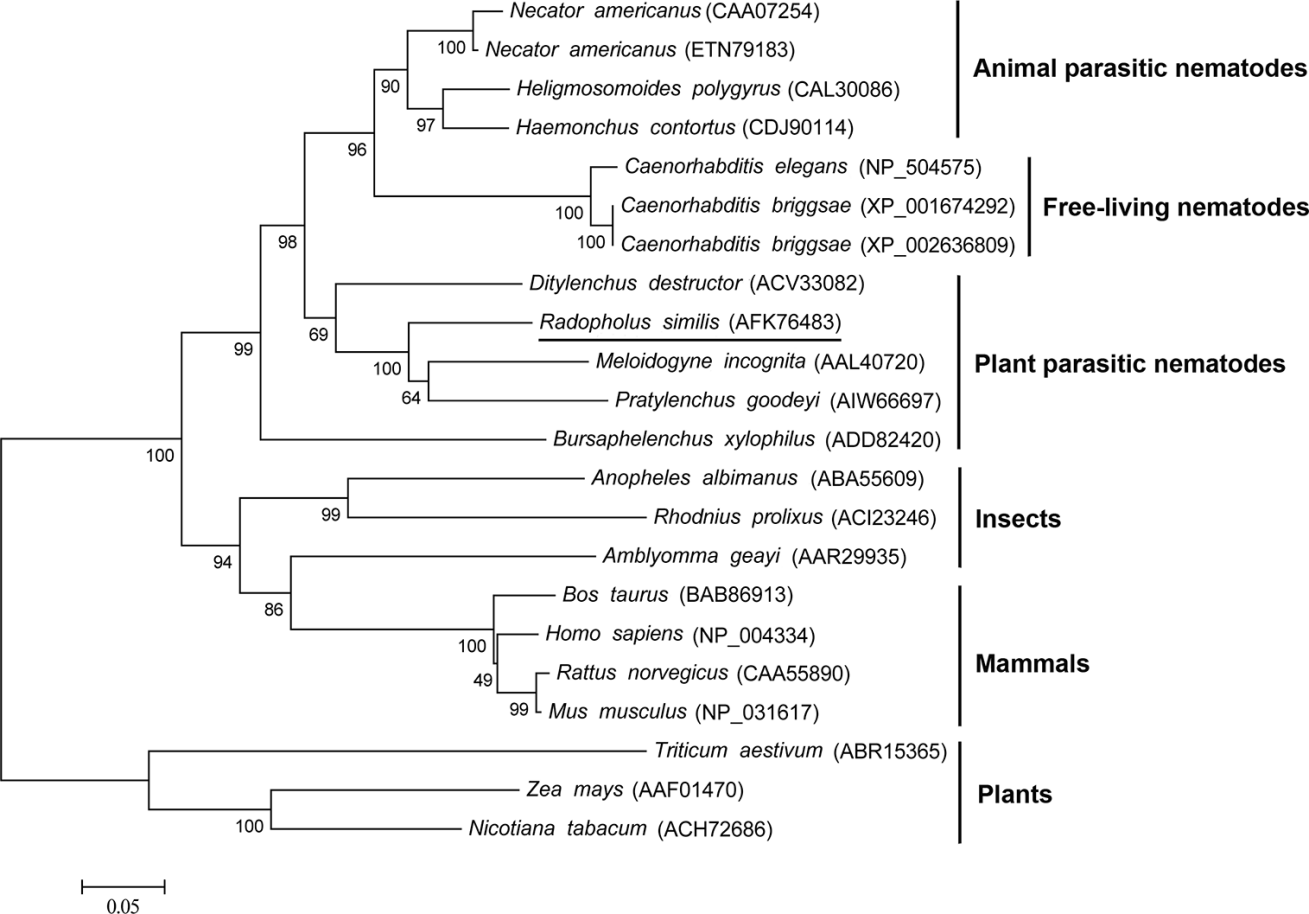
Neighbor-joining phylogenetic tree of CRT proteins. The phylogram was constructed according to the amino acid sequences of 22 CRT proteins from 20 different species using MEGA 5.0. *R*. *similis* CRT is underlined. The accession numbers of the sequences are shown in brackets.

A phylogenetic tree was constructed based on the aa sequences of 22 CRT proteins from 20 different species (Fig 2). *Rs*-CRT from *R*. *similis*, *Mi*-CRT from *M*. *incognita* and *Pg*-CRT from *P*. *goodeyi* were grouped on the same branch, suggesting that they have a closer phylogenetic relationship. In addition, *Rs*-CRT and the other 21 CRT proteins were divided into 6 groups: animal parasitic nematodes, free-living nematodes, plant parasitic nematodes, insects, mammals, and plants.

**Fig 3:**
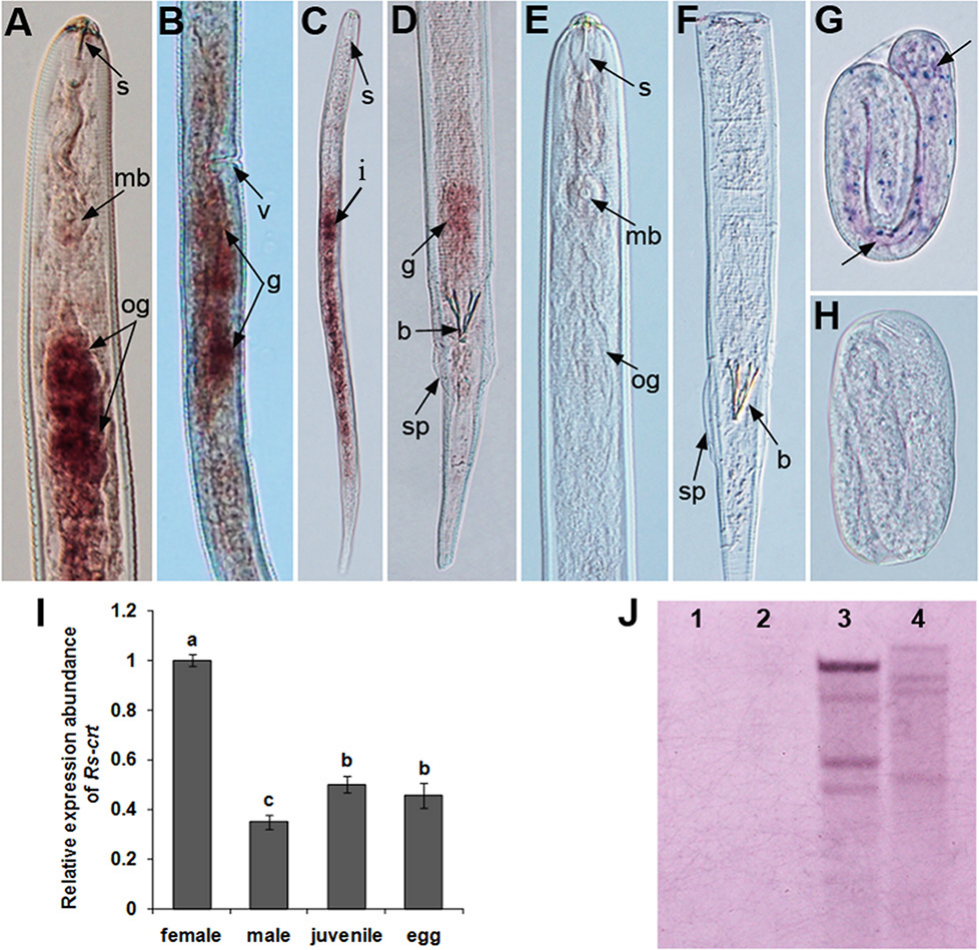
Localization, expression and Southern blot analysis of *Rs-crt* in *Radopholus similis*. (A-H) Tissue localization of *Rs-crt* mRNA via *in situ* hybridization. *Rs-crt* was localized in the oesophageal glands (A) and gonads (B) of females, the gonads of males (D), the intestines of juveniles (C) and the eggs (G). (E, F and H) No hybridization signal was observed in nematodes hybridized with the DIG-labelled sense RNA probe. b, bursa; g, gonads; mb, medium bulb; og, oesophageal glands; s, stylet; sp, spicules; v, vulva. (I) *Rs-crt* expression in females, males, juveniles and eggs of *R*. *similis*. Bars indicate the standard errors of the mean data (n = 3), and different letters indicate significant differences (*p*<0.05) between treatments. (J) Southern blot analysis of *Rs-crt*. Lanes 1–2, gDNA from carrot callus digested with *EcoR* I and *Xba* I; lanes 3–4, gDNA from *R*. *similis* digested with *EcoR* I and *Xba* I.

Southern blotting was carried out to investigate the gene copy number of *Rs-crt* in *R*. *similis*. The 378-bp-long DIG-labelled probe hybridized to multiple fragments in both the *EcoR*I- and *Xba*I-digested gDNA of *R*. *similis*. No hybridization signal was detected when using carrot callus gDNA. Neither the genomic coding region nor the cDNA contained an *EcoR*I or *Xba*I site. These results indicate that *Rs-crt* exist as a multi-copy gene in the *R*. *similis* genome (Fig 3J).

### Tissue localization and expression of *Rs-crt* in *R*. *similis*

*In situ* hybridization was performed to localize *Rs-crt* expression. A DIG-labelled antisense probe specifically hybridized with *Rs-crt* transcripts in the oesophageal glands and gonads of females (Fig 3A and 3B), the gonads of males (Fig 3D), the intestines of juveniles and the eggs of *R*. *similis* (Fig 3C and 3G). No hybridization signal was observed in the nematodes when the control sense probe was used (Fig 3E, 3F and 3H). The qPCR results showed that the *Rs-crt* mRNA transcript was present in all developmental stages of *R*. *similis*, and the expression was significantly highest in females (*p* < 0.05). Compared to its level in females, percentage reduction of *Rs-crt* expression were 65.1%, 50.0% and 54.5% in males, juveniles and eggs, respectively. *Rs-crt* expression in males was significantly lower (*p* < 0.05) than that in juveniles and eggs. No significant difference in expression (*p* > 0.05) was observed between juveniles and eggs (Fig 3I).

### Induction of RNAi in *R*. *similis* by soaking with target-specific *Rs-crt* dsRNA

**Fig 4:**
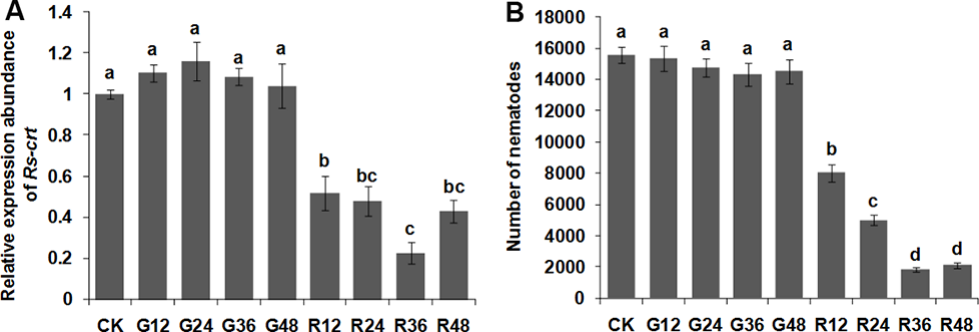
Inducing RNAi in *Radopholus similis* by soaking with *Rs-crt* dsRNA. (A) Expression of *Rs-crt* mRNA in *R*. *similis* treated with *Rs-crt* dsRNA. Bars indicate the standard errors of the mean (n = 3). (B) Number of nematodes on carrot disks 50 d after the inoculation of 30 females. Bars indicate the standard errors of the mean data (n = 5). CK: untreated nematodes; G12-G48: nematodes treated with *egfp* dsRNA for 12 h, 24 h, 36 h and 48 h, respectively; R12-R48: nematodes treated with *Rs-crt* dsRNA for 12 h, 24 h, 36 h and 48 h, respectively. Different letters indicate significant differences (*p*<0.05) between treatments.

qPCR was employed to measure the *Rs-crt* silencing efficiency in *R*. *similis* after treatment with *Rs-crt* dsRNA for 12 h, 24 h, 36 h and 48 h, and it was shown that *Rs-crt* expression was decreased significantly (*p* < 0.05) by 53.0%, 58.9%, 79.0% and 58.5%, respectively, compared to that observed in the corresponding *egfp* dsRNA treatments. The silencing efficiency was enhanced with increasing incubation durations within a certain range and was highest at 36 h. The e*gfp* dsRNA-treated and untreated nematodes showed no significant difference (*p* > 0.05) in *Rs-crt* expression (Fig 4A). After being cultured on carrot disks for 50 d, *R*. *similis* treated with *Rs-crt* dsRNA for 12 h, 24 h, 36 h and 48 h had significantly lower reproduction (*p* < 0.05) than untreated and e*gfp* dsRNA-treated nematodes. Nematodes treated with *Rs-crt* dsRNA for 36 h had the lowest reproduction, showing significant differences (*p* < 0.05) compared with the 12 h and 24 h treatments. There was no significant difference (*p* > 0.05) in reproduction among the untreated and e*gfp* dsRNA-treated nematodes (Fig 4B).

**Fig 5:**
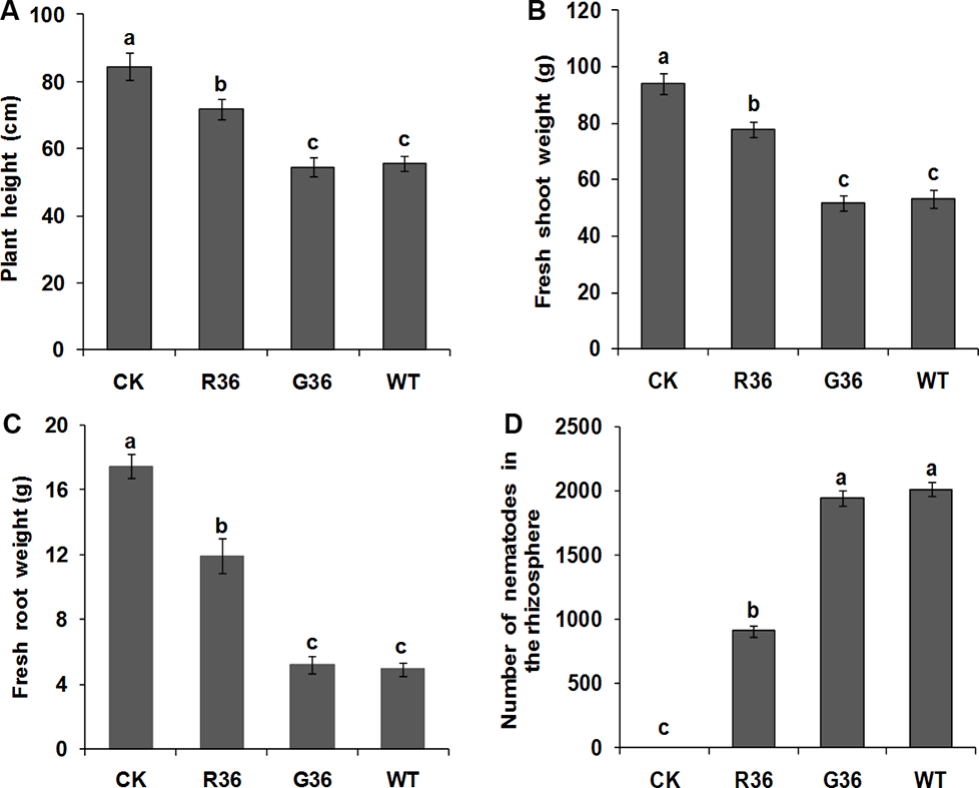
The pathogenicity of *Radopholus similis* to tomato plants is decreased significantly after soaking with *Rs-crt* dsRNA. Plant height (A), fresh shoot weight (B), fresh root weight (C) and number of nematodes in the rhizosphere (D) of plants at 60 d after nematodes inoculation. CK: uninoculated healthy plants; R36 and G36: plants inoculated with nematodes treated with *Rs-crt* dsRNA and e*gfp* dsRNA for 36 h, respectively; WT: plants inoculated with untreated nematodes. Bars indicate the standard errors of the mean data (n = 5), and different letters indicate significant differences (*p* <0.05) between treatments.

At 60 d after inoculation, the tomato plants inoculated with nematodes treated with *Rs-crt* dsRNA for 36 h showed significant increases in growth parameters compared with plants inoculated with e*gfp* dsRNA-treated nematodes for 36 h and plants inoculated with untreated nematodes (i.e.,the two control treatments); however, the values of these growth parameters were still significantly lower than those in uninoculated healthy plants (*p* < 0.05) (Fig 5A–5C). The number of nematodes in the rhizosphere of plants inoculated with nematodes treated with *Rs-crt* dsRNA for 36 h was significantly lower (*p* < 0.05) than that in the two control treatments (Fig 5D). There was no significant difference between the two control groups (*p* > 0.05) in terms of the three growth parameters or nematode numbers (Fig 5). These results confirmed that the pathogenicity of *R*. *similis* was significantly reduced after treatment with *Rs-crt* dsRNA for 36 h.

### Production and molecular analysis of transgenic RNAi plants

The constructed RNAi expression vector pFGC-RS-crt2 contained a CaMV35S promoter, 407-bp sense and antisense fragments of *Rs-crt* cDNA, a CHSA intron and an OCS terminator. The inverted repeats present in *Rs-crt* were separated by the CHSA intron (S4 Fig). Transgenic tomato plants were regenerated from transformed calli, which were selected based on resistance to kanamycin (S5 Fig). Using this approach, 27 *Rs-crt* transgenic plants, 18 *egfp* transgenic plants and 13 empty vector transgenic plants were obtained. These transgenic lines did not show obvious morphological differences compared with wild-type tomato plants (S5 Fig).

**Fig 6:**
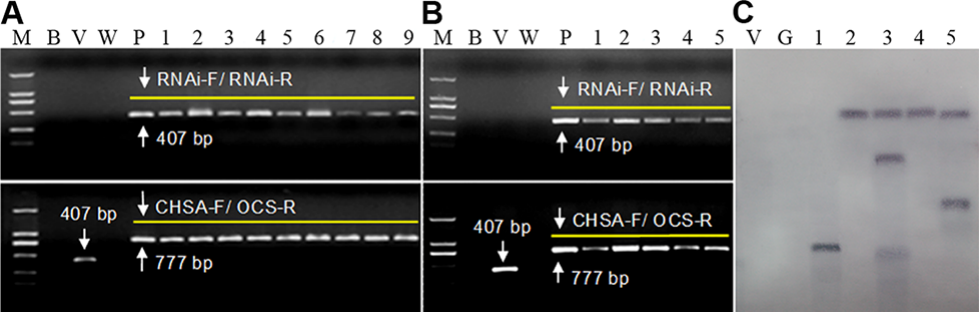
Molecular analysis of transgenic tomato plants. (A) Independently derived transgenic lines were detected via PCR using the primers RNAi-F/RNAi-R and CHSA-F/OCS-R (lanes 1–9: independent *Rs-crt* transgenic lines). (B) Independent transgenic lines were detected via RT-PCR using the primers RNAi-F/RNAi-R (lanes 1–5: RNA from *Rs-crt* transgenic lines 1, 2, 3, 4 and 5). M, DNA marker (DL2000); B, blank control without template; V, empty transformation vector plant; W, wild-type plant (negative control); P, positive plasmid control. (C) Southern blot analysis of *Nde*I-digested gDNA from T0 transgenic plant leaves (lanes v and g: gDNA from empty transformation vector plants and e*gfp* transgenic plants (controls); lanes 1–5, gDNA from *Rs-crt* transgenic lines 1, 2, 3, 4 and 5).

Two fragments with lengths of 407-bp and 777-bp were amplified from the DNA of T0 *Rs-crt* transgenic tomato plants. A 407-bp fragment was also amplified from the empty transformation vector plants, but no bands were amplified from the wild-type plants (Fig 6A). Two fragments with lengths of 315-bp and 685-bp were amplified from the *egfp* transgenic plants (results not shown). These results revealed that the hairpin dsRNAs were successfully inserted into the tomato gDNA. Southern blot analysis showed that PCR-positive *Rs-crt* transgenic plants carried 1–3 copies of the target coding sequence. Conversely, no hybridization band was observed when using gDNA from the *egfp* transgenic plants and empty transformation vector plants (Fig 6C). RT-PCR analysis indicated that the integrated *Rs-crt* dsRNA was successfully expressed in transgenic plants (Fig 6B). The ratio of positive and negative T1 *Rs-crt* transgenic plants (Nos. 1, 2 and 4) was 3:1, and a 407-bp fragment corresponding to the *Rs-crt* sequence was amplified from positive plants. These results revealed that the integrated *Rs-crt* could be stably inherited in transgenic tomato gDNA (S6 Fig). Previous reports have shown that the effectiveness of RNAi is higher in single-copy lines than in other lines [52, 53]. Therefore, we selected the single-copy *Rs-crt* transgenic plant (No. 2) for further analyses.

### T2 generation transgenic tomato plants expressing *Rs-crt* dsRNA exhibit greater resistance to *R*. *similis*

**Fig 7:**
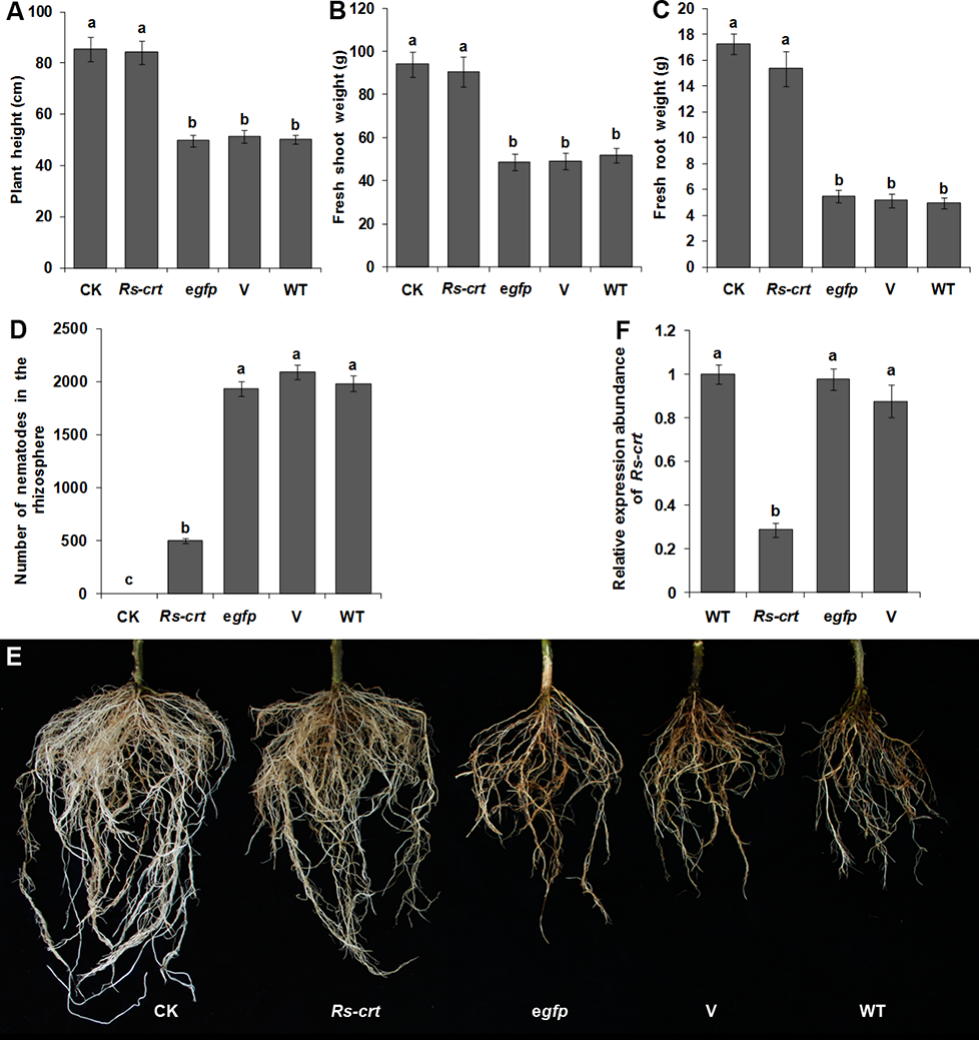
T2 transgenic tomato plants expressing *Rs-crt* dsRNA showed improved resistance to *Radopholus similis*. Plant height (A), fresh shoot weight (B), fresh root weight (C), number of nematodes in the rhizosphere (D) and root infection symptoms (E) in different plants at 60 d after inoculation with 1,000 nematodes. Bars indicate the standard errors of the mean (n = 5). (F) qPCR assay to detect *Rs-crt* expression in *R*. *similis* individuals collected from T2 transgenic plants. Bars indicate the standard errors of the mean (n = 3). CK, uninoculated wild-type plants; *Rs-crt*, *Rs-crt* transgenic plants; e*gfp*, e*gfp* transgenic plants; V, empty transformation vector plants; WT, wild-type plants. Different letters indicate significant differences (*p*<0.05) between treatments.

At 60 d after inoculation, the growth parameters of *Rs-crt* transgenic plants (No. 2) were significantly (*p* < 0.05) increased compared to the growth parameters of three types of inoculation control plants (e*gfp* transgenic plants, empty transformation vector plants and wild-type tomato plants); however, no significant difference (*p* > 0.05) was observed between the *Rs-crt* transgenic plants and the uninoculated wild-type plants. The percentage reduction of plant height, fresh shoot weight and fresh root weight of *Rs-crt* transgenic plants were only 1.64%, 3.63% and 10.92%, respectively, compared to the uninoculated wild-type tomato plants (CK), (Fig 7A–7C). The number of nematodes in the rhizosphere of the *Rs-crt* transgenic plants was significantly lower (*p* < 0.05) than that observed in the rhizosphere of the three inoculation control plants (Fig 7D). Additionally, the degree of root damage was much lower and there was no obvious root rot in the *Rs-crt* transgenic plants compared with the three inoculation control plants (Fig 7E). There was no significant difference (*p* > 0.05) in these pathogenicity measures among the three types of inoculation control plants, and their roots were severely damaged, showing obvious root rot (Fig 7). The inoculation tests demonstrated that resistance to *R*. *similis* was significantly increased in *Rs-crt* transgenic plants.

### *Rs-crt* expression is significantly suppressed in *R*. *similis* feeding on T2 *Rs-crt* transgenic tomato plants

The percentage reduction of *Rs-crt* expression in *R*. *similis* fed on T2 *Rs-crt* transgenic plants were 70.6%, 67.3% and 71.3% compared with that in nematodes fed on the e*gfp* transgenic plants, empty transformation vector plants and wild-type tomato plants, respectively. There were no significant differences (*p* > 0.05) among the three inoculation control treatments (Fig 7F). Therefore, it can be concluded that the suppression of *Rs-crt* expression in *R*. *similis*, which was caused by feeding on the roots of *Rs-crt* dsRNA-expressing transgenic tomato plants, resulted in a reduction of pathogenicity.

### Persistence and inheritance of *Rs-crt* silencing induced by *in vitro* RNAi and plant-mediated RNAi

**Fig 8:**
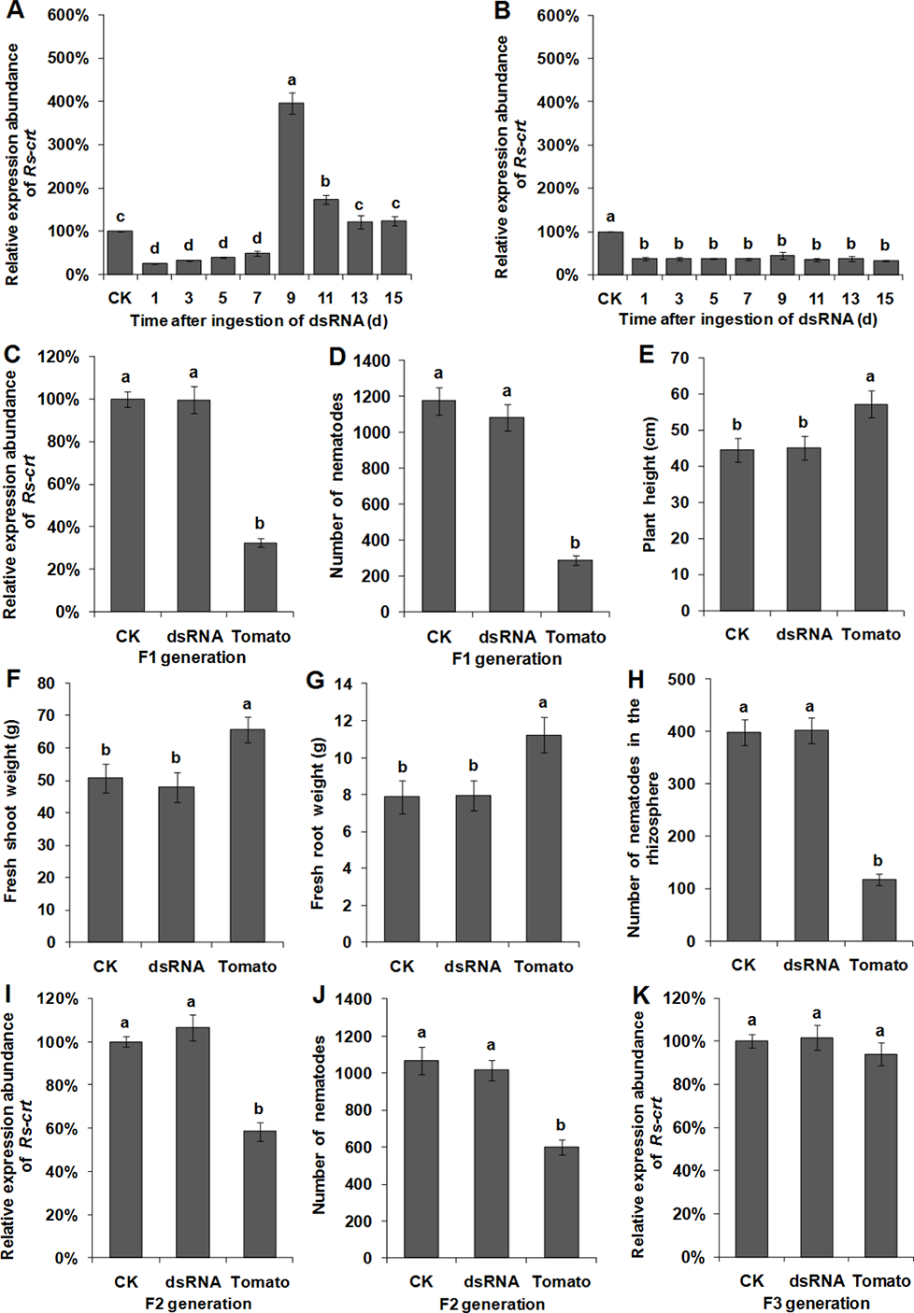
Persistence and inheritance of *Rs-crt* silencing induced by RNAi. The recovery of *Rs-crt* expression in *Radopholus similis* at different times after soaking in *Rs-crt* dsRNA for 36 h (A) and in *R*. *similis* collected from T2 *Rs-crt* transgenic tomato roots (B). 1–15, *Rs-crt* expression levels in nematodes maintained in water and sampled at 1, 3, 5, 7, 9, 11, 13 and 15 d post-treatment, respectively. qPCR assay to detect *Rs-crt* expression in F1 (C), F2 (I) and F3 (K) nematodes. Bars indicate the standard errors of the mean (n = 3). Number of F1 (D) and F2 (J) nematodes on carrot disks at 30 d after the inoculation of 30 females. Plant height (E), fresh shoot weight (F), fresh root weight (G) and the number of nematodes in the rhizosphere (H) at 45 d after the plants were inoculated with 200 F1 nematodes. CK, untreated nematodes; dsRNA, nematodes treated with *Rs-crt* dsRNA for 36 h; Tomato, nematodes collected from T2 *Rs-crt* transgenic tomato roots. Bars indicate the standard errors of the mean (n = 5), and different letters indicate significant differences (*p*<0.05) between different treatments.

Following recovery in sterile water for 1–7 d, *Rs-crt* expression in *R*. *similis* soaked with *Rs-crt* dsRNA for 36 h was significantly (*p* < 0.05) reduced by 50.6–73.9% compared with *Rs-crt* expression in untreated nematodes (CK group); however, no significant difference (*p* > 0.05) was found between the different recovery times. After recovery for 9 d and 11 d, *Rs-crt* expression was significantly (*p* < 0.05) increased to 396% and 174% of that in the CK group, respectively. After recovery for 13 d and 15 d, *Rs-crt* expression returned to normal levels (Fig 8A). However, *Rs-crt* expression in nematodes isolated from T2 *Rs-crt* transgenic tomato plants and recovered in water for 1–15 d was significantly (*p* < 0.05) reduced by 55.2%-66.8% compared with the CK group, but no significant difference (*p* > 0.05) was found between the different recovery times (Fig 8B).

*Rs-crt* expression in F1 nematodes derived from T2 *Rs-crt* transgenic plants was reduced by 67.4% compared with the CK group. This reduction was significantly greater (*p* < 0.05) than that observed in the *Rs-crt* dsRNA soaking treatment and CK groups; however no significant difference (*p* > 0.05) was observed between the latter two groups (Fig 8C). After being cultured for 30 d, the F1 nematodes derived from *Rs-crt* transgenic plants exhibited significantly lower reproduction than the F1 nematodes derived from the *Rs-crt* dsRNA soaking treatment and CK groups (*p* < 0.05), but there was no significant difference between the latter two groups (*p* > 0.05) (Fig 8D). At 45 d after inoculation, the growth parameters of wild-type tomato plants inoculated with F1 nematodes derived from *Rs-crt* transgenic plants were significantly increased compared to the *Rs-crt* dsRNA soaking treatment and CK groups, and the number of nematodes in the rhizosphere was 118, which was significantly lower than that in the *Rs-crt* dsRNA soaking treatment (402) and CK groups (398) (*p* < 0.05). However, there was no significant difference between the last two groups (*p* > 0.05) (Fig 8E–8H).

*Rs-crt* expression in F2 nematodes derived from T2 *Rs-crt* transgenic plants was significantly (*p* < 0.05) reduced by 41.6% compared with the CK group (Fig 8I). After being cultured for 30 d, the F2 nematodes derived from *Rs-crt* transgenic plants exhibited significantly lower (*p* < 0.05) reproduction compared with the F2 nematodes derived from the *Rs-crt* dsRNA soaking treatment and CK groups (Fig 8J). *Rs-crt* expression in the F3 nematodes derived from T2 *Rs-crt* transgenic plants showed no significant difference (*p* > 0.05) compared with the *Rs-crt* expression in F3 nematodes derived from the *Rs-crt* dsRNA soaking treatment and CK groups (Fig 8K).

These results suggested that *Rs-crt* expression in *R*. *similis* isolated from T2 *Rs-crt* transgenic plants was significantly inhibited after recovery in water and that this inhibition was still observable in the F1 and F2 nematodes. The reproductive capability and pathogenicity of the F1 and F2 nematodes were significantly reduced (*p* < 0.05). However, *Rs-crt* expression in F3 nematodes returned to normal levels. Conversely, *in vitro* RNAi-induced *Rs-crt* silencing only lasted for a limited time and could be recovered; the F1 and F2 nematodes exhibited normal expression of *Rs-crt* and showed normal reproductive capacity and pathogenicity. Overall, these results indicate that plant-mediated RNAi*-*induced *Rs-crt* silencing could be effectively transmitted to F2 nematodes.

## Discussion

This study is the first to describe the cloning of the full-length *Rs-crt* sequence from *R*. *similis* and the identification of its structure, features and function. We confirmed that *Rs-crt* plays key roles in the reproduction and pathogenicity of *R*. *similis* using *in vitro* and plant-mediated RNAi. Plant-mediated RNAi-induced *Rs-crt* silencing could be effectively transmitted to F2 nematodes, whereas the silencing effect of *Rs-crt* induced by *in vitro* RNAi was no longer detectable in F1 and F2 nematodes.

Park *et al*. [34] reported that *crt-1* was expressed in the pharynx, intestine, body-wall muscles, coelomocytes and sperm of *C*. *elegans*. Khalife *et al*. [28] confirmed that *crt* was mainly expressed in the genital organs and digestive duct epithelia of *Schistosoma mansoni*. Gao *et al*. [33] demonstrated that *crt* was ubiquitously expressed in different tissues and at different developmental stages of *Haemaphysalis qinghaiensis*. In plant parasitic nematodes, *Mi-crt* is expressed in the female gonads and in the subventral oesophageal glands of *M*. *incognita* [39]. The present study revealed that the expression and localization of *Rs-crt* are associated with the biological functions of CRT. CRT and other related proteins are necessary for *R*. *similis* to break the host defence response and complete the infection process as well as to obtain nutrients for metabolism and reproduction. *R*. *similis* females are responsible for both infection and reproduction; consequently, *Rs-crt* expression was found to be highest in females. CRT plays important roles in infection, the establishment of the parasitic relationship, reproduction and cell differentiation in nematodes [36–39]. The successful invasion of host plants and establishment of the parasitic relationship by *R*. *similis* is a precondition for other functions to be implemented; therefore, *Rs-crt* expression was observed to be slightly higher in juveniles than in eggs, but this difference was not significant. *R*. *similis* males, which present a degraded stylet and oesophagus, are non-parasitical and are not necessary for reproduction, as this species can reproduce via parthenogenesis. Therefore, *Rs-crt* expression was found to be lowest in males. Cheng *et al*. [13] reported that the *Ab-far-1* expression level was lowest in males and was only 4.7% of the *Ab-far-1* expression level in females. In the current study, the *Rs-crt* expression level was also lowest in males and was 34.9% of the *Rs-crt* expression level in females. This result reveals that CRT is a multifunctional protein that also plays important roles in *R*. *similis* males. Previous studies have shown that CRT plays key roles in protein export, cell adhesion, mRNA degradation and cellular calcium homeostasis [39]. *Mi-crt* from *M*. *incognita* and *Rs-eng-1b* from *R*. *similis* are genes that are expressed in the oesophageal gland and help nematodes to break the host defence, establish the parasitic relationship, and digest host tissue to obtain necessary nutrients [12, 39]. In this study, *Rs-crt* was found to be localized in the oesophageal glands of *R*. *similis*; therefore, it is likely to have functions related to *Mi-crt* and *Rs-eng-1b*. Additionally, *Rs-crt* was found to be localized in the reproductive system, possibly because CRT plays roles in reproduction, development and cell differentiation in *R*. *similis*. A previous report showed that *Bx-crt-1* has functions related to *B*. *xylophilus* development and reproduction [38]. Similarly, the present study revealed that silencing of *Rs-crt* through *in vitro* RNAi significantly reduced the reproductive capability and pathogenicity of *R*. *similis*. These results were consistent with the expression and localization patterns of *Rs-crt* described above.

In this study, we confirmed that *Rs-crt* expression was significantly inhibited in *R*. *similis* that feed on *Rs-crt* transgenic tomato plants, and the pathogenicity of the nematodes was significantly reduced on transgenic plants expressing *Rs-crt* dsRNA. These results were consistent with the roles of *Rs-crt* in *R*. *similis*, which were validated by *in vitro* RNAi. Plant-mediated RNAi has been used to validate gene functions in cyst nematodes, root-knot nematodes and other sedentary endoparasites [23–26, 54]. As there is limited information available regarding *in planta* RNAi against migratory endoparasites [55] compared to sedentary endoparasites [21–26], the current investigation will enrich the database of RNAi against migratory endoparasites.Rosso *et al*. [11] reported that the *in vitro* RNAi-induced silencing effect was temporary, and transcript depletion of *mi-crt* and *Mi-pg-1* was undetectable 68 h after soaking. The expression of *hg-eng-1* in *Heterodera glycines* (J2) was severely inhibited 16 h after forced ingestion of dsRNA; however, this expression level was significantly increased after recovery in water for 10 d and returned to normal levels after recovery for 15 d [14]. The present study revealed that *Rs-crt* expression in *R*. *similis* soaked with *Rs-crt* dsRNA for 36 h was significantly inhibited after recovery in water for 1–7 d; however, *Rs-crt* expression was significantly increased to 3.96-fold of that in the control group after 9 d of recovery and returned to normal levels after 13 d of recovery. These results indicate that *in vitro* RNAi-induced gene silencing is time-limited, possibly because the amount of ingested dsRNA is limited and its effect decrease as dsRNA degrades in nematodes. Following treatment with dsRNA, the expression levels of the target geneswere highest in *R*. *similis* and *H*. *glycines* after recovery for 9 d and 10 d, respectively. This result may have occurred because the regulation system of nematodes triggers an increase in mRNA synthesis when the expression of the corresponding target gene is low for an extended period of time after the loss of RNAi efficiency. Subsequently, the expression levels return to normal levels under the control of standard gene regulation mechanisms.

Steeves *et al*. [22] reported that the number of *H*. *glycines* eggs was significantly reduced after the nematodes were fed on transgenic soybean roots expressing specific MSP dsRNA, and ability of the progeny (F1) to successfully reproduce was also impaired. These results provided the first confirmation of the inheritability of gene silencing induced by plant-mediated RNAi in sedentary plant nematodes; however, the question of whether the progeny of migratory plant nematodes can inherit this RNAi effect has not yet been addressed. The present study revealed that *Rs-crt* expression in F1 and F2 *R*. *similis* derived from T2 *Rs-crt* transgenic tomato plants was severely inhibited, and the reproductive capability and pathogenicity of the nematodes was significantly reduced. The effect of RNAi treatment disappeared in F3 nematodes. Conversely, *in vitro* RNAi-induced *Rs-crt* silencing lasted only for a limited time, and *Rs-crt* expression could be recovered. Thus, we confirmed that plant-mediated RNAi-induced *Rs-crt* silencing could be effectively inherited by F2 *R*. *similis*, whereas the silencing effects induced by *in vitro* RNAi were not heritable. These results suggest that plant-mediated RNAi could overcome the limitations of *in vitro* RNAi and might be successfully applied in both sedentary and migratory plant nematodes. These findings provide a scientific basis for further studies of the relationships between nematodes and their hosts and for the development of new methods for controlling plant parasitic nematodes and other pests.

## Supporting Information

S1 Fig The partial cDNA sequence of the *Radopholus similis* calreticulin gene. An 832-bp PCR fragment was amplified using the degenerate primers Cal1Fand Cal1R.
(TIF)

S2 Fig *Rs-crt* cDNA (GenBank accession number AFK76483) and predicted amino acid sequences. The 5′- and 3′- untranslated regions (UTR) are shown in lowercase letters, and the open reading frame is shown in uppercase letters. The putative polyadenylation signal (attaaa) is boxed. ATG, initiation codon; TGA, stop codon.
(TIF)

S3 Fig Genomic sequence of the *Radopholus similis* calreticulin gene. The *Rs-crt* genomic coding region contains six introns and seven exons. Introns are marked in dark grey. ATG, initiation codon; TGA, stop codon.
(TIF)

S4 Fig Construction of the plant RNAi vector expressing hairpin *Rs-crt* dsRNA in transgenic tomato plants. The constructed pFGC-RS-crt2 vector contains a CaMV 35S promoter, 378-bp sense and antisense fragment of *Rs-crt* cDNA, a CHSA intron and an octopine synthase (ocs) terminator.
(TIF)

S5 Fig Generation and morphology of transgenic tomato plants. (A-E) Development of transgenic plants expressing *Rs-crt* dsRNA. (A) Preculture of explants. (B) Putative transformed calli growing on selection medium. (C) Transgenic plantlets germinated from transformed calli. (D, E) Transgenic plants growing on rooting medium. (F) Growth morphology of the transgenic tomato leaves. No obvious differences were observed between the transgenic and wild-type tomato plants. 1, 2, 3 and 4: expression of wild-type, *Rs-crt* transgenic, e*gfp* transgenic and empty transformation vector tomato leaves, respectively.
(TIF)

S6 Fig Genetic stability analysis of T1 generation *Rs-crt* transgenic plants. **(**A-C) Expression of genomic DNA from *Rs-crt* transgenic lines 1, 2 and 4, respectively; M: DNA marker (DL2000); 1–15: independent T1 *Rs-crt* transgenic lines from the same T0 transgenic tomato seeds.
(TIF)
