# Supplementary figures and images for: A Nematode Calreticulin, Rs-CRT, Is a Key Effector in Reproduction and Pathogenicity of Radopholus similis
Source: PLoS One. 2015 Jun 10;10(6):e0129351. doi: 10.1371/journal.pone.0129351 (PMC4465493; doi:10.1371/journal.pone.0129351)

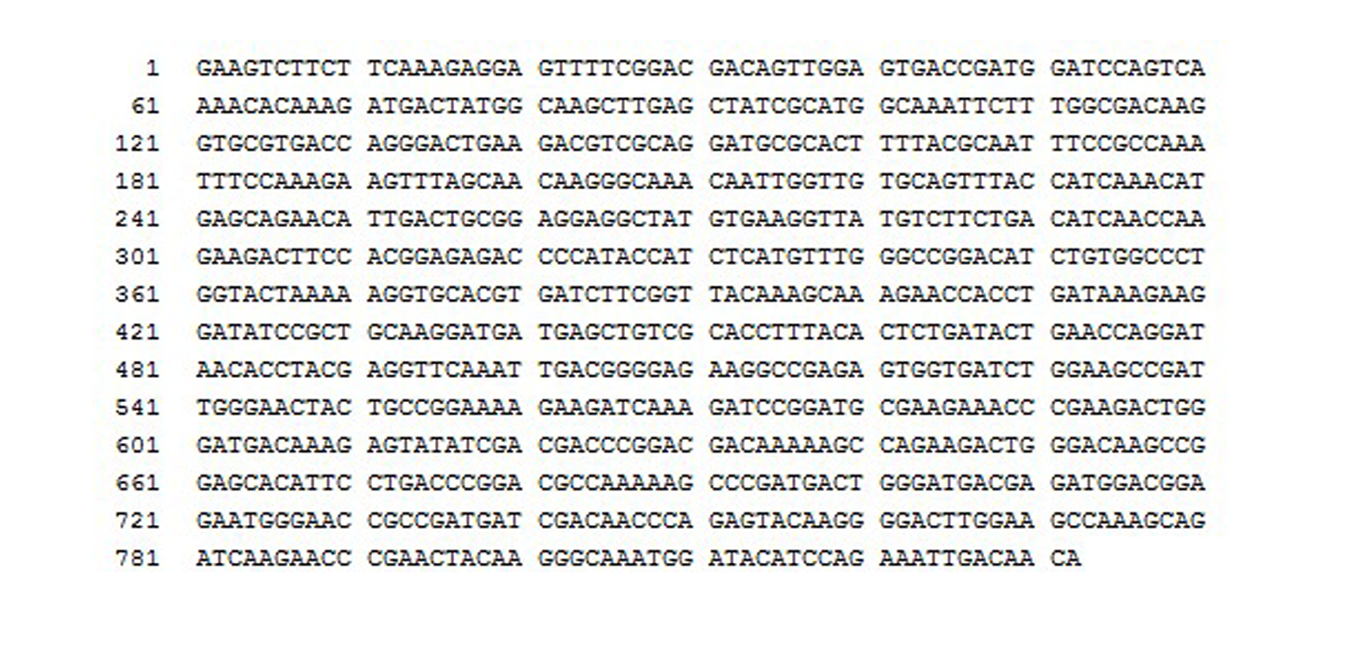

Supplement: S1 Fig — An 832-bp PCR fragment was amplified using the degenerate primers Cal1Fand Cal1R. (TIF) [file pone.0129351.s001.tif]

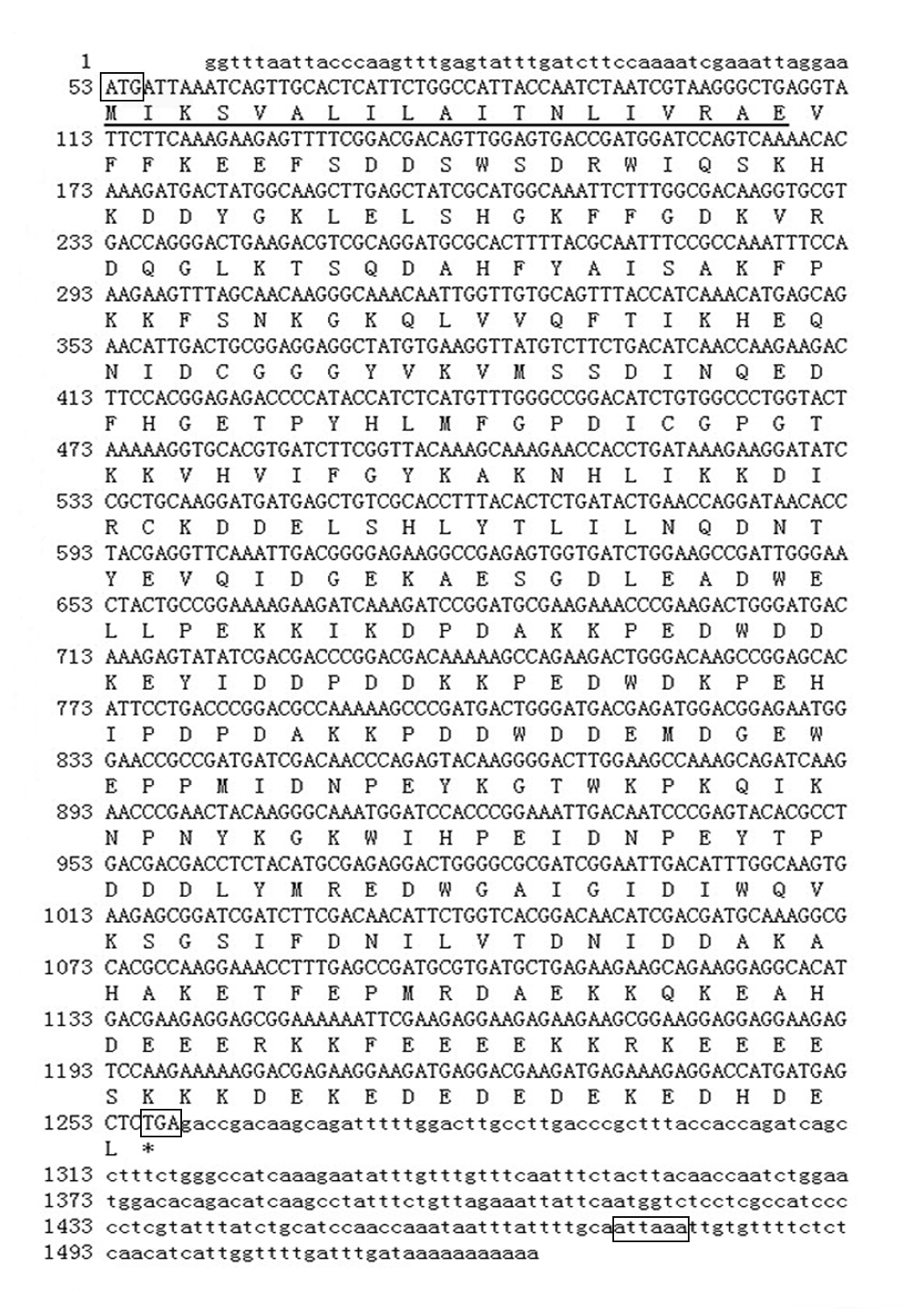

Supplement: S2 Fig — The 5′- and 3′- untranslated regions (UTR) are shown in lowercase letters, and the open reading frame is shown in uppercase letters. The putative polyadenylation signal (attaaa) is boxed. ATG, initiation codon; TGA, stop codon. (TIF) [file pone.0129351.s002.tif]

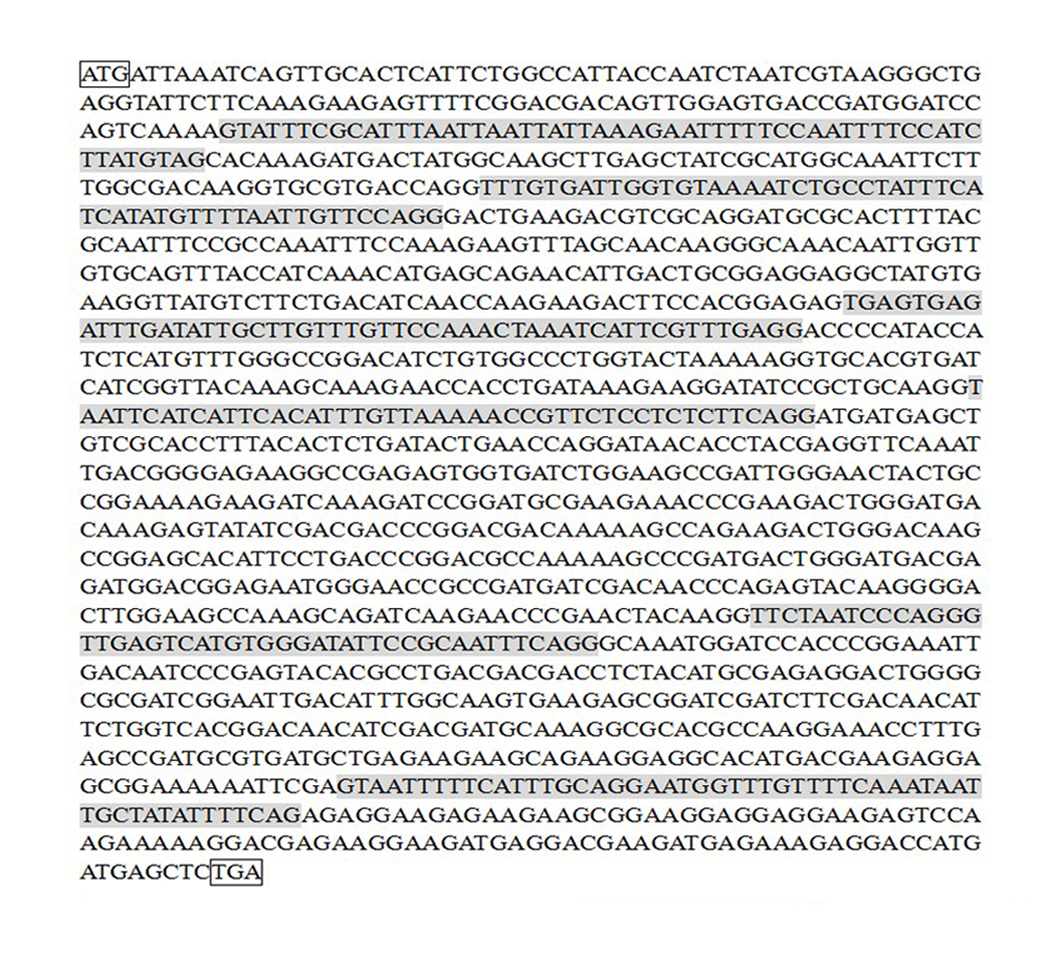

Supplement: S3 Fig — The Rs-crt genomic coding region contains six introns and seven exons. Introns are marked in dark grey. ATG, initiation codon; TGA, stop codon. (TIF) [file pone.0129351.s003.tif]

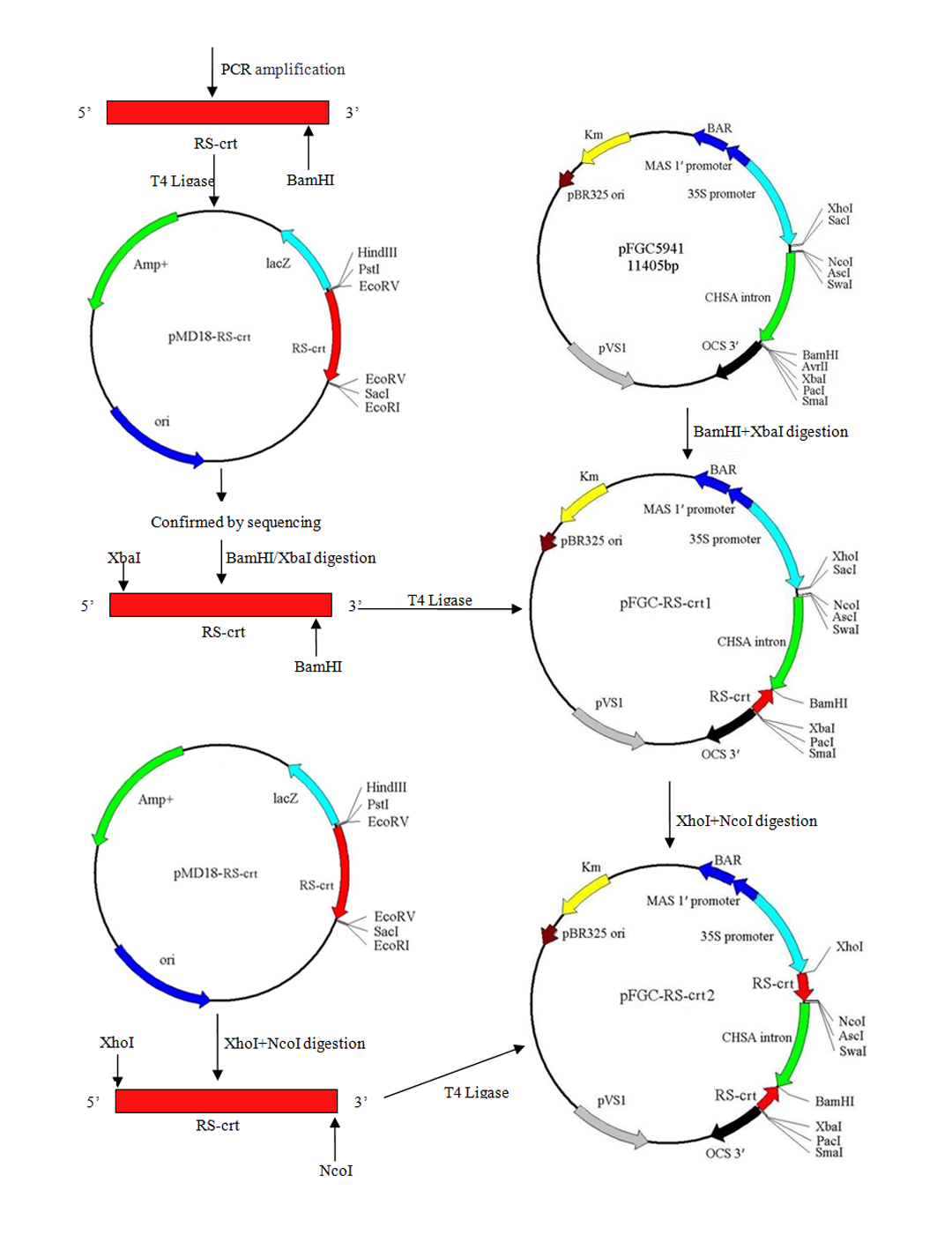

Supplement: S4 Fig — The constructed pFGC-RS-crt2 vector contains a CaMV 35S promoter, 378-bp sense and antisense fragment of Rs-crt cDNA, a CHSA intron and an octopine synthase (ocs) terminator. (TIF) [file pone.0129351.s004.tif]

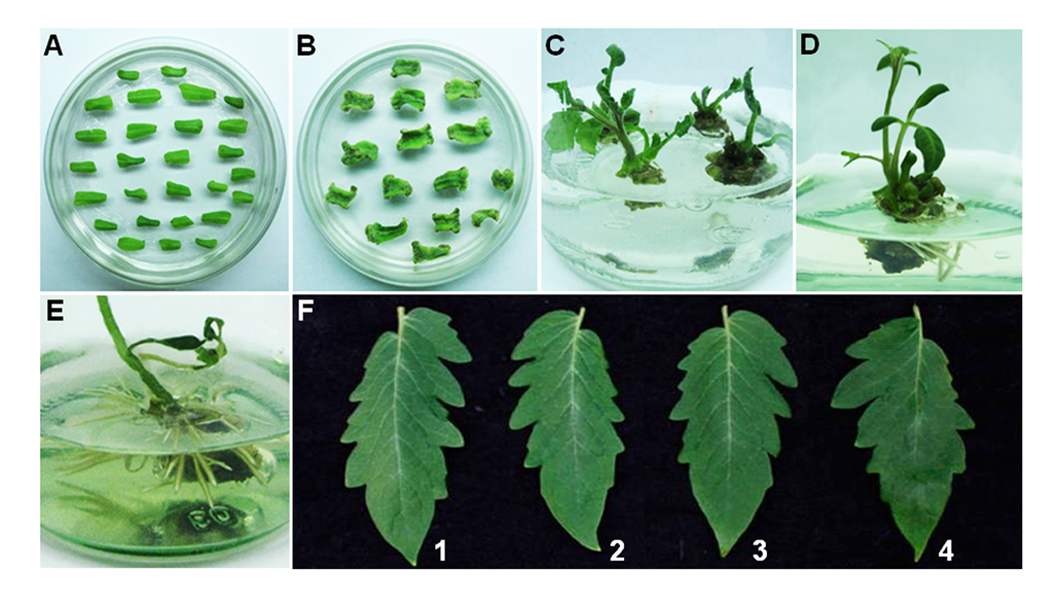

Supplement: S5 Fig — (A-E) Development of transgenic plants expressing Rs-crt dsRNA. (A) Preculture of explants. (B) Putative transformed calli growing on selection medium. (C) Transgenic plantlets germinated from transformed calli. (D, E) Transgenic plants growing on rooting medium. (F) Growth morphology of the transgenic tomato leaves. No obvious differences were observed between the transgenic and wild-type tomato plants. 1, 2, 3 and 4: expression of wild-type, Rs-crt transgenic, egfp transgenic and empty transformation vector tomato leaves, respectively. (TIF) [file pone.0129351.s005.tif]

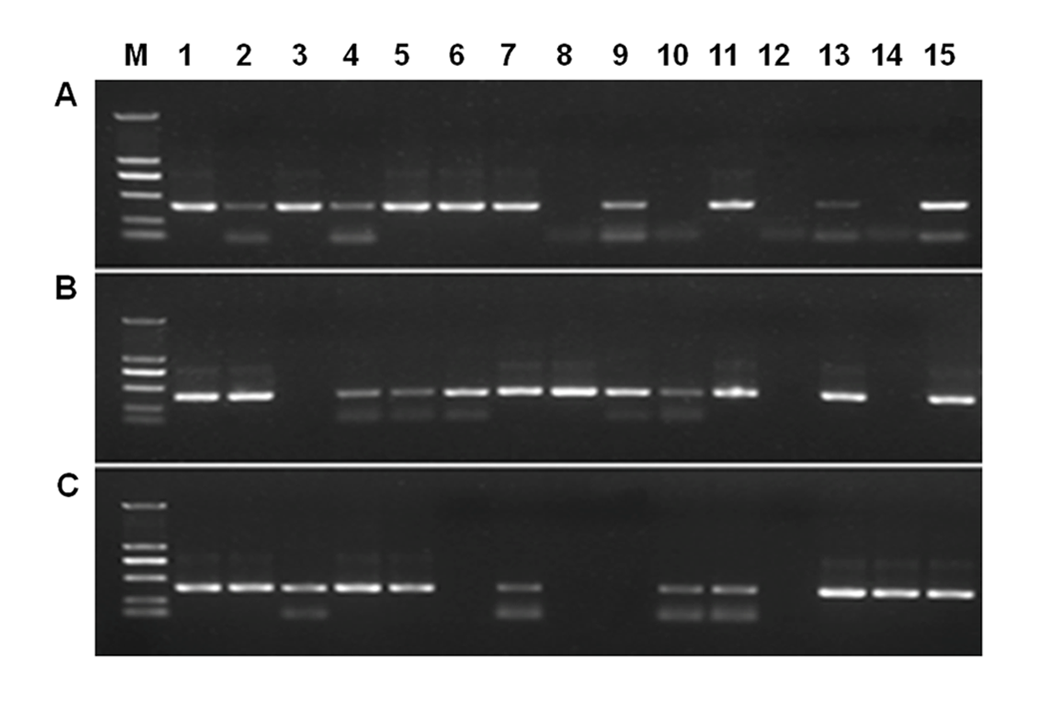

Supplement: S6 Fig — (A-C) Expression of genomic DNA from Rs-crt transgenic lines 1, 2 and 4, respectively; M: DNA marker (DL2000); 1–15: independent T1 Rs-crt transgenic lines from the same T0 transgenic tomato seeds. (TIF) [file pone.0129351.s006.tif]
